# Enzyme-free optical DNA mapping of the human genome using competitive binding

**DOI:** 10.1093/nar/gkz489

**Published:** 2019-06-05

**Authors:** Vilhelm Müller, Albertas Dvirnas, John Andersson, Vandana Singh, Sriram KK, Pegah Johansson, Yuval Ebenstein, Tobias Ambjörnsson, Fredrik Westerlund

**Affiliations:** 1 Department of Biology and Biological Engineering, Chalmers University of Technology, Gothenburg, Sweden; 2 Department of Astronomy and Theoretical Physics, Lund University, Lund, Sweden; 3 Clinical Chemistry, Sahlgrenska University Hospital, Gothenburg, Sweden; 4 School of Chemistry, Center for Nanoscience and Nanotechnology, Center for Light-Matter Interaction, Raymond and Beverly Sackler Faculty of Exact Sciences, Tel Aviv University, Tel Aviv, Israel

## Abstract

Optical DNA mapping (ODM) allows visualization of long-range sequence information along single DNA molecules. The data can for example be used for detecting long range structural variations, for aiding DNA sequence assembly of complex genomes and for mapping epigenetic marks and DNA damage across the genome. ODM traditionally utilizes sequence specific marks based on nicking enzymes, combined with a DNA stain, YOYO-1, for detection of the DNA contour. Here we use a competitive binding approach, based on YOYO-1 and netropsin, which highlights the contour of the DNA molecules, while simultaneously creating a continuous sequence specific pattern, based on the AT/GC variation along the detected molecule. We demonstrate and validate competitive-binding-based ODM using bacterial artificial chromosomes (BACs) derived from the human genome and then turn to DNA extracted from white blood cells. We generalize our findings with *in-silico* simulations that show that we can map a vast majority of the human genome. Finally, we demonstrate the possibility of combining competitive binding with enzymatic labeling by mapping DNA damage sites induced by the cytotoxic drug etoposide to the human genome. Overall, we demonstrate that competitive-binding-based ODM has the potential to be used both as a standalone assay for studies of the human genome, as well as in combination with enzymatic approaches, some of which are already commercialized.

## INTRODUCTION

Optical DNA mapping (ODM) is based on visualizing the sequence of ultralong DNA molecules (>100 000 basepairs (bp)), covering length scales on DNA that are not accessible with modern sequencing techniques (1). This opens up the possibility to identify large structural variations (2–11), as well as to use optical maps as scaffolds to organize sequencing contigs along complex genomes (12–15). Optical maps are created by labeling individual DNA molecules in a sequence-specific manner, stretching the molecules, and imaging them using a fluorescence microscope (16). The stretching is done either on a glass surface (17–19) or in nanofluidic channels (16,20–22), where the latter allows for more uniform stretching while still permitting high-throughput analysis.

Most examples of ODM in the literature are based on enzymatic labeling, as pioneered by Schwartz and colleagues (23). At present, nicking enzymes are most widely used, which create a nick in the DNA backbone at a specific (7 bp) sequence (13,24,25). The nick is then repaired with a polymerase and a ligase in presence of fluorescently labelled nucleotides. The result is an array of sequence-specific ‘dots’ along DNA that can for example be matched to a genome of interest to find structural variations (2,3), or used for *de novo* assembly of complex genomes (12,26,27). Issues with nick-labeling include <100% labeling efficiency and that nicking makes the DNA more fragile, where two nicks occurring too close together on opposite strands of the DNA will cause a double strand break, leading to shorter DNA molecules and loss of information. Also, regions without any recognition sites remain uncharacterized. Recently, enzymatic labeling with methyltransferases was introduced (28,29). This strategy positions labels on DNA without damaging the DNA, which opens up the possibility for denser labeling.

Enzymatic assays can also be used to label other features along the DNA. Examples include the epigenetic marks methylation (30,31) and hydroxymethylation (32,33), as well as DNA damage (34). By labeling the DNA with multiple colors, it is possible to combine sequence specific labels with epigenetic DNA marks, which makes it possible to, for example, locate hydroxymethylations along the human genome (33).

**Figure 1.**
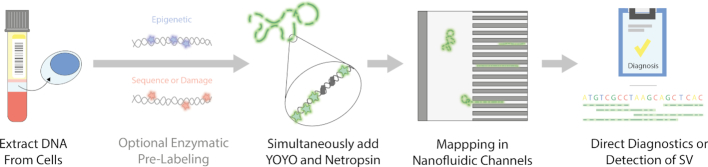
Schematic overview of the optical DNA mapping assay. Large DNA fragments are extracted from the cells of interest prior to the one step labeling of the DNA with YOYO-1 and netropsin. The DNA is then stretched in nanofluidic channels and imaged using a fluorescence microscope. Prior to creating the affinity-based barcode with YOYO-1 and netropsin, there is a possibility to pre-label the DNA with either epigenetic, sequence, or damage specific fluorescent marks. The obtained data can be used for multiple applications, such as diagnostics of genetic diseases, detection of structural variations (SV), de novo assembly of complex genomes and/or detection of other genetic or epigenetic marks.

We have developed an ODM assay based on the binding of two small molecules, YOYO-1 and netropsin, to DNA. Netropsin blocks AT-rich regions from binding of the fluorescent, nonspecific, YOYO-1, which leads to that AT-rich regions are darker than GC-rich regions (Figure 1) (35). We have previously used this competitive binding-based assay for studies on bacterial DNA (36), and in particular bacterial plasmids (37–42). The principle is the same as the melt mapping assay initially proposed by Reisner *et al.* (43), where DNA is stained with YOYO-1 and partially melted. Melt mapping has been used on the human DNA in several studies (44–46) where very long DNA molecules (>1 mega base pair (Mb)) are stretched one by one in two counter propagating flows. This principle requires extensive hands on manipulation and is not suited for high-throughput applications.

All the enzymatic labeling protocols require extensive sample preparation with, among other things, several washing steps to remove unbound fluorescent dyes. The competitive binding-based method, on the other hand, requires only one step of simultaneous addition of YOYO-1 and netropsin to DNA. No additional step to remove excess YOYO-1 is necessary since YOYO-1 is only fluorescent when bound to DNA (47). Furthermore, in most enzymatic labeling protocols, YOYO-1 is added to visualize the DNA backbone and to estimate the length of each fragment. Using the competitive binding assay, the images of YOYO-1 labeled DNA will also provide sequence information and the possibility to locate a specific fragment along the human genome, without increasing the demands on the optical setup. A final benefit with the affinity-based approaches is that they do not depend on the presence of specific enzymatic sites along the DNA, instead providing continuous sequence information along the DNA.

In this study we have, for the first time, applied competitive binding-based ODM to human DNA, focusing on investigating the possibilities for future high-throughput analysis. We establish the basic principles using bacterial artificial chromosomes (BACs, 123–203 kilobases (kb) in size) and later turn to DNA extracted from peripheral blood mononuclear cells (PBMCs), where we show that we can map long DNA molecules (∼200–600 kb) to the human genome with high precision. We generalize our findings *in silico* by showing that we can in theory cover 97% of the human genome with a fragment size of 333 kb. Finally, we demonstrate the possibility of combining competitive binding with enzymatic labelling by locating DNA damage sites induced by the cytotoxic drug etoposide in human PBMCs along the genome. The latter is, to our knowledge, the first demonstration of combining affinity-based and enzymatic labeling in ODM.

## MATERIALS AND METHODS

### Samples and DNA extraction

Bacterial Artificial Chromosomes (BACs) were used to optimize the experimental settings and the *P*-value threshold (37,39). For details about the five BACs used in the study, see Supplementary Table S1. The circular BACs were linearized using the restriction enzyme NotI to remove the cloning vector. To that end, 600 ng BAC was incubated with 20 U of restriction enzyme (NotI-HF, New England BioLabs) at 37°C for 1 h in 1× CutSmart Buffer (New England BioLabs) with a total volume of 20 μl. The reaction was terminated by heat deactivation at 65°C for 20 min.

Human DNA was extracted from excess blood with healthy blood counts, obtained from the Hematology unit at the Clinical Chemistry Department at Sahlgrenska University Hospital, Sweden. Peripheral blood mononuclear cells (PBMCs) were isolated from the blood samples through gradient centrifugation using Lymphoprep (Axis-Shield PoC AS) according to the manufacturer's instructions. The harvested cells were resuspended in Cell Suspension Buffer from the CHEF Mammalian Genomic DNA Plug Kit (BIO-RAD). The cell suspension was mixed with 2% CleanCut Agarose (BIO-RAD) to obtain a final concentration of 0.75% agarose and the mixture was left for solidification in 100 μl plug molds at 4°C for 15 min. The solidified agarose plugs were incubated with Proteinase K overnight at 50°C according to the BIO-RAD protocol. Next, the plugs were washed four times in 1× Wash Buffer (BIO-RAD) for 1 h at room temperature with gentle agitation. The plugs were further washed in 1 mM phenylmethylsulfonyl fluoride followed by a final wash with 0.5× Wash Buffer. The agarose plugs were digested by first melting the plugs at 70°C for 10 min, followed by incubation with 0.5 U of Agarase (Thermo Fisher Scientific) at 42°C for 1 h. The DNA concentration was determined using a Nanodrop 1000 (Thermo Scientific).

For DNA damage experiments, the PBMCs were treated with the topoisomerase II inhibitor etoposide. In brief, 100 mM stock solutions of etoposide (Sigma) were prepared in DMSO and stored at −80°C. The cells were resuspended in RPMI medium 1640 at 4 × 10^6^/0.5 ml and incubated with 37.5 μM etoposide under dark conditions at 37°C for 2 h. The cells were washed by addition of 1× PBS containing 0.01% bovine serum albumin and centrifugation at 200 g for 2 min at 4°C and the supernatant was discarded. The washing step was repeated twice. DNA extraction was then carried out as described above.

### DNA labeling

The sequence specific DNA barcode was obtained by a single-step staining reaction using YOYO-1 (ex. 491 nm/em. 509 nm, Invitrogen) and netropsin (Sigma-Aldrich) (35). The DNA was incubated at 50°C for 30 min in a 10 μl solution containing 1 μM DNA sample, 1 μM λ-DNA (used as internal size reference, 48 502 bp, Roche Biochem Reagents), 0.2 μM YOYO-1 and 60 μM netropsin in 0.5× TBE (Tris-Borate-EDTA, Medicago) resulting in molar ratios of 1:10 (YOYO-1: DNA base pair) and 1:300 (YOYO-1: Netropsin). The solution was then diluted ten times with 88 μl ultrapure water and 2 μl β-mercaptoethanol (BME, Sigma-Aldrich), to a final buffer concentration of 0.05× TBE. BME was used to suppress photodamage to the sample.

For DNA extracted from cells treated with etoposide, DNA damage sites were labeled prior to staining the DNA with YOYO-1 and netropsin. The labeling reaction, adapted form Zirkin *et al.* (34), was performed using 500 ng of DNA, 10 U DNA Pol 1, 100 μM of dATP, dGTP, dCTP, 10 μM dTTP and 10 μM Aminoallyl-dUTP-ATTO-647N (ex. 643 nm/em. 665 nm, Jena Bioscience), in 1× nick translation buffer (0.05 M Tris–HCl pH 7.2, 0.1 mM dithiothreitol, 0.01 M MgSO4, 50 μg/ml BSA) in a final volume of 100 μl. The incubation was done overnight at 16°C and the reaction was terminated with 2.5 μl of 0.25 M EDTA.

### Nanofluidic experiments

Nanofluidic devices were fabricated in silica using standard methods, as described elsewhere (20). The device consists of two pairs of loading wells, each pair connected via a microchannel. The two separate microchannels are in turn connected by 120 nanochannels with dimensions of 100 × 150 nm^2^ and a length of 500 μm. The chips were pre-wetted with 0.05× TBE buffer (2% BME v/v) to achieve uniform conditions prior to sample loading. A sample volume of 10 μl was added to one of the loading wells on the chip and buffer in the others and the DNA was loaded into the nanochannels using pressure driven N_2_ flow.

Imaging was performed using an inverted fluorescence microscope (Zeiss AxioObserver.Z1) equipped with a 63× (1.6× optovar) oil immersion objective (NA = 1.46, Zeiss), a Colibri 7 light source (Zeiss) and an Andor iXon EMCCD Camera. In total, a series of up to 100 images were acquired for each molecule using an exposure time of 100 ms and a FITC filter set (Zeiss). For the dual channel imaging of etoposide damaged DNA, a total of 20 frames were acquired in each channel, using 1× optovar and a dual bandpass 90 HE filter set (Zeiss).

### Data analysis

The data analysis for mapping individual DNA molecules to the human genome was performed using application specific Matlab code, for details see Supplementary Methods. In short, DNA molecules are individually detected from each time frame and stacked as kymographs, where each row represents the intensity along the DNA molecule in one single frame. The features in the kymographs are then aligned to compensate for the small thermal fluctuations of the DNA inside the nanochannels. When only a single time frame was used, no alignment was done. Next, the aligned kymographs are collapsed, rendering a time averaged 1D intensity trace along the extension of the DNA molecule, referred to as a barcode, that can be compared to the theoretical barcode of the human genome. For the initial comparisons of the BACs with the human genome, averaged experimental barcodes (consensus barcodes) from multiple copies of the same BACs were used (38).

To compare an experimental barcode to the human genome, theoretical barcodes based on the base pair sequence of all 24 chromosomes (GRCh38.p7 dataset, retrieved from NCBI) were generated, using previously established methods (36,37). In short, the theoretical barcode is estimated based on sequence-dependent binding constants of netropsin, a sequence-independent binding constant of YOYO-1, and the point-spread function of the microscope. We do not take the minor variations in the physical properties of DNA, such as the small sequence dependence of the persistence length (48), or effects of YOYO-1 binding (49), into account. The theoretical barcode was adjusted based on the degree of DNA extension for each experiment, *i.e*. the number of base pairs per pixel, that was determined using the internal size reference λ-DNA (48 502 bp), or directly calculated based on the size of a known BAC sequence. The best fit to the human genome was assessed using a match score, based on a Pearson correlation coefficient (*C*), evaluating each possible start position (pixel), for both the original, as well as the flipped, version of the experimental barcode, compared to the theoretical human genome, where the highest match score (*C*_max_) corresponds to the best fit. In the case of the BACs, for which the correct positions on the theoretically generated human genome were known, all experimental barcodes with *C*_max_ within a range of 20 pixels (2 × 10^−7^ fraction of human genome) were classified as being correctly placed.

For the experiments where DNA damage sites were visualized at the same time as obtaining the sequence specific barcode, the images obtained from the ATTO-647 channel were processed separately. Prior to extracting the intensity trace along the extension of the DNA molecule, retrieved from the YOYO-1 channel, the raw stack of images from the ATTO-647 channel of each individual DNA molecule was averaged. The intensity trace along the region of interest (ROI) was extracted by averaging over 5 pixels. The background was then removed by applying a Gaussian filter (with standard deviation of 8 pixels) and subtracting the intensity obtained when averaging over 5 pixels above the ROI. Finally, the background corrected intensity trace was normalized.

## RESULTS AND DISCUSSION

In this study we demonstrate how optical DNA maps, i.e. barcodes, obtained via a single-step competitive binding assay, can be mapped with high precision to the human genome. A schematic representation of the assay is shown in Figure 1. The barcode is created by simultaneously adding YOYO-1 and netropsin to DNA (35). In a typical experiment ∼500 pg of DNA is loaded into a nanofluidic chip. By stretching the DNA molecules in nanochannels, the sequence specific barcode is read using a fluorescence microscope (see Materials and Methods for details). We optimize the assay on BACs of known sequence, before applying it on human DNA extracted from blood samples, including experiments were the barcode is combined with enzymatic labeling of DNA damage.

### Evaluation and optimization of performance using bacterial artificial chromosomes

**Figure 2.**
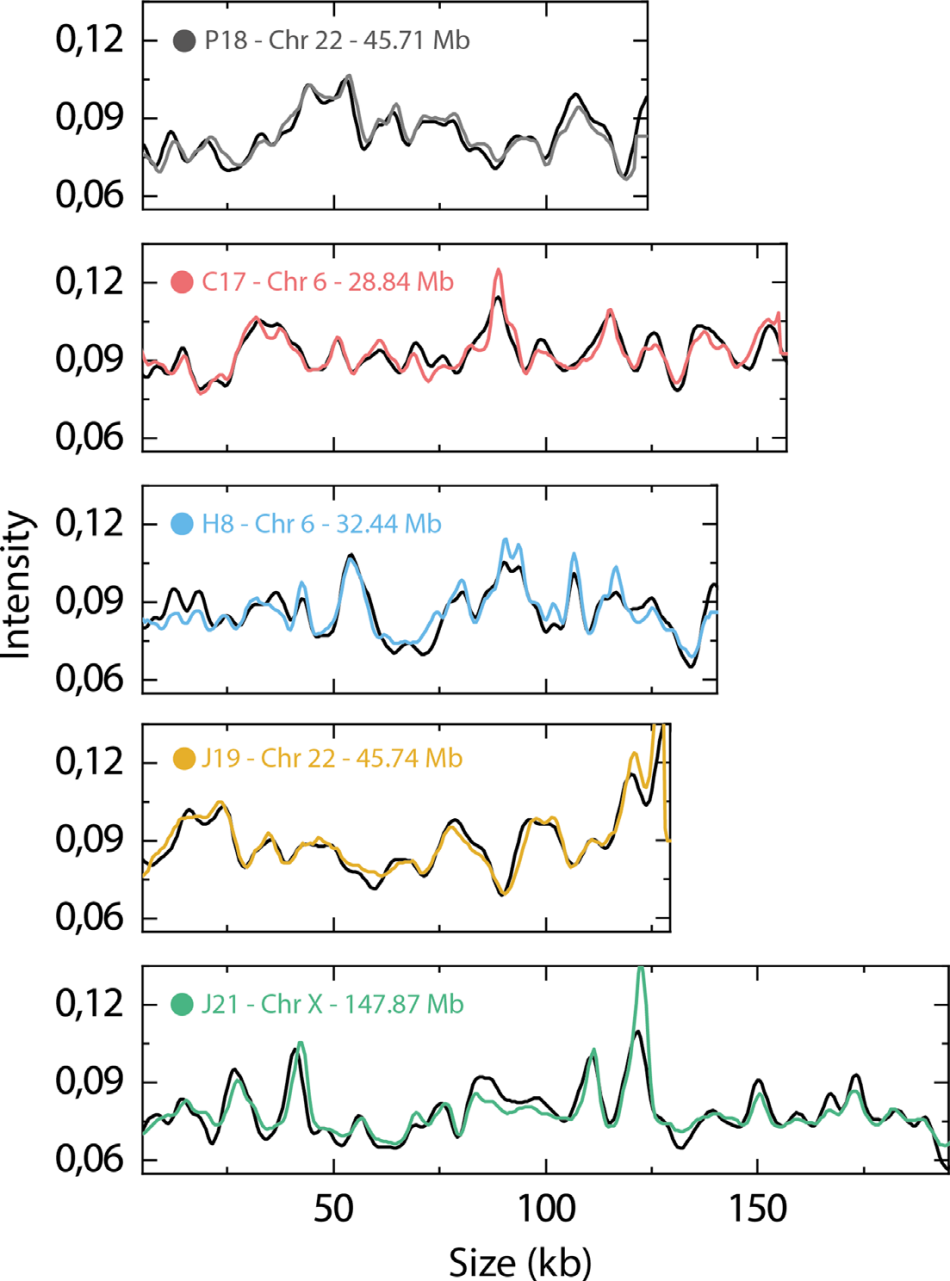
Fits of averaged experimental BAC barcodes to the human genome. Each averaged experimental BAC barcode is shown at the position of the best obtained fit along the human genome. The text in each plot displays BAC name followed by chromosome number as well as position along that chromosome.

Five different BACs were used to assess the potential of the assay, as well as to optimize the experimental and computational parameters. As a first evaluation of applying competitive binding based ODM on the human genome, multiple copies (see Supplementary Table S1 for details) of experimental barcodes (each consisting of 10 frames) for each BAC were averaged in order to maximize the signal to noise ratio (SNR) of the experimental data (38). The averaged experimental barcodes were then scanned against a theoretically generated barcode of the entire human genome (see Methods), based on the base pair sequence (36,37), in order to find the position of the best fit (Figure 2).

The averaged experimental barcodes for all five BACs obtain the highest *C*_max_ to the correct position of the human genome and display an excellent overlap between the experimental and theoretical barcode, demonstrating the high discriminatory power of the barcodes. However, when applying the assay to the entire human genome, it will not be possible to average multiple experimental barcodes in order to increase the SNR, due to random fragmentation of the DNA. Also, to optimize the throughput of the assay, the number of time frames acquired for each individual molecule should be minimized.

The number of frames acquired for each individual molecule will affect both the throughput, increasing with decreasing number of frames, as well as the accuracy, increasing with increasing number of frames. To investigate the relation between the accuracy (quantified by *C*_max_) and the number of time frames used, multiple experimental barcodes for each of the five BACs were compared to their corresponding theoretical barcode, varying the number of time frames. The results (Supplementary Figure S1A) show how *C*_max_ increases with increasing number of frames. Supplementary Figure S1B demonstrates how the noise is reduced when averaging multiple time frames, increasing the similarity with the theoretical barcode as the number of time frames increases. For the experiments below, the number of frames was fixed to ten, where ∼96% of the maximal *C*_max_ was obtained. It should be noted that even with a single time frame, the average *C*_max_ is still as high as 83% of its maximal value. Hence, depending on application, the optimal number of time frames will vary, balancing throughput and accuracy.

When an experimental barcode is compared to the theoretical barcode of the human genome, the number of bp per pixel, reflecting the degree of DNA extension in the nanofluidic channels, is needed. We use λ-DNA as an internal size reference to convert between pixels and base-pairs to minimize the error due to variations in DNA stretching between different experiments. Due to experimental uncertainties, there is however still a need to account for small variations in the extension of the DNA to make sure that as many of the experimental barcodes as possible find their correct position on the human genome. Increasing the allowed size variation will increase the probability of fitting to the correct position on the human genome, but at the same time increases the ‘risk’ of the experimental barcode fitting well to other regions of the human genome. Experimental barcodes of the five different BACs, all consisting of ten frames, were compared to the human genome with different size variations allowed (Supplementary Figure S2). The optimal amount of stretching for the five examined BACs was always between 2 and 10%, with an overall optimal value of 5%.

### Establishment of a *P*-value threshold

**Figure 3.**
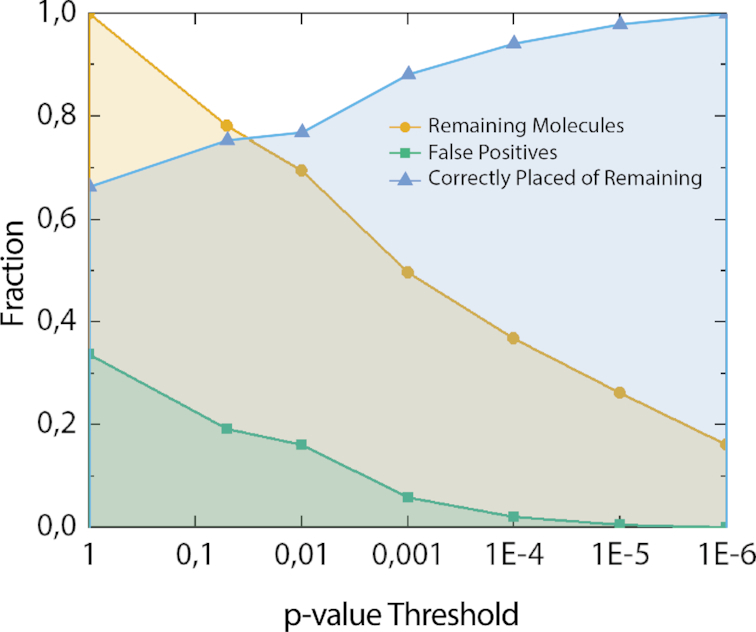
Evaluation of *P*-value threshold using BACs. Remaining molecules (yellow) after applying different *P*-value thresholds, and fraction of false positives (green), of experimental barcodes from BACs when mapped to the human genome. Blue line is fraction of correctly placed experimental barcodes out of the remaining (yellow) molecules at each *P*-value threshold. All barcodes (*N* = 187) were compared using 10 frames and up to 5% stretch (1% steps).

When comparing the experimental fragments to the human genome it is important to distinguish correctly placed barcodes from false positives. To eliminate false positives, a *P*-value was introduced, based on the obtained *C*_max_ score and the size of each individual fragment (see Supplementary Methods for details). The effect of decreasing *P*-value thresholds on the number of false positives can be seen in Figure 3, and examples of fits of individual BAC molecules to their correct positions in the human genome in Supplementary Figure S3.

As expected, the number of molecules passing the *P*-value threshold decreases with a decreasing *P*-value threshold, with below 20% left at *P* = 10^−6^ (Figure 3). However, as the *P*-value threshold was decreased to 10^−6^, false positives were completely eliminated, and hence only barcodes that match to the correct position along the human genome remained. The need for a lower *P*-value threshold compared to our previous studies (37,38), can to some extent be explained by the sequence similarities found in the human genome, that leads to that experimental barcodes match with a high score (*C*_max_) not only to one position, but rather two or more. This is not the case for the randomized reference barcodes used to generate the *P*-values. Therefore, a very low threshold is necessary to make sure that the barcodes indeed fit only to the correct position. The *P*-value is very sensitive to the size of the experimental fragments. For the largest BAC, J21 (203 kb), 53% of the data passed the *P*-value threshold of 10^−6^ compared to only 11% for the smallest BAC P18 (123 kb). A longer fragment contains more information, and the number of highly similar sequences in the human genome will decrease rapidly with increasing size. Since *C*_max_ for a match between an experimental and theoretical barcode is not sensitive to size, mapping longer DNA molecules will make it possible to use a significantly larger fraction of the data at *P* = 10^−6^, than for the shorter BAC molecules.

### Mapping DNA extracted from blood samples

**Figure 4.**
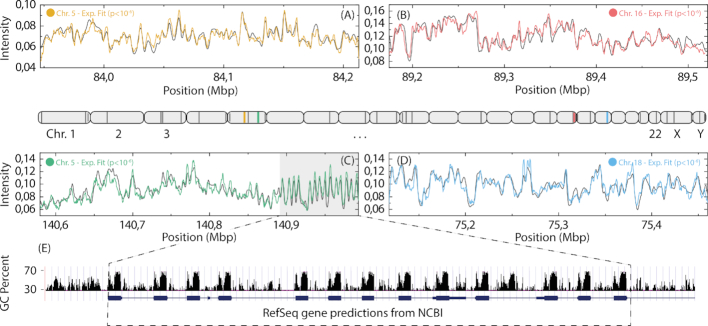
Fits of experimental barcodes of single DNA molecules to the human genome. Four examples (**A**–**D**) of experimental barcodes mapped to the human genome (10 frames, 5% stretch, 1% steps). The light gray area in (C) highlights a repetitive sequence which is shown in more detail in (**E**). The approximate positions of the fits for all DNA molecules passing the *P*-value threshold (*P*< 10^−6^) are shown as dark gray lines (colored lines mark positions for barcodes shown in the figure) on a schematic illustration of the human genome (center). (E) RefSeq genes (blue) and GC content (black) for the repetitive region found in (C). The GC sequence content is displayed with a 5bp window.

To evaluate the applicability of matching any DNA fragments to the human genome, DNA was extracted from PBMCs. The DNA extraction was performed in agarose plugs to obtain long (>200 kb) DNA molecules (see Methods for details). All experimental barcodes (each consisting of 10 frames) were mapped to the human genome, using a *P*-value threshold of 10^−6^. Examples of fits between experimental barcodes and the human genome reference barcode are shown in Figure 4.

The experimental and theoretical barcodes showed excellent overlap to the identified location within the human genome, all with *P*-values well below 10^−6^. For the barcode in Figure 4C, a highly repetitive region was observed (light gray area, details in Supplementary Figure S4). The repeats (Figure 4E) correspond to the GC rich exons of the Protocadherin Alpha gene cluster which is composed of 15 cadherin superfamily genes related to the mouse CNR genes. These neural cadherin-like cell adhesion proteins are integral plasma membrane proteins that most likely play a critical role in the establishment and function of specific cell-cell connections in the brain (50,51). The observation of this repetitive region highlights the ability of ODM to acquire long range sequence information, which is a great advantage compared to traditional sequencing techniques. Using ODM, repeats can be quantified directly from the barcode, and variations between different cells identified, information which is likely to be averaged out when using DNA sequencing alone.

In total, 95% (36/38) of the DNA molecules collected (215–439 kb in size, average 333 kb) passed the *P*-value threshold of 10^−6^, compared to 16% (30/187) for the BACs. As expected, the longer DNA fragments extracted from the PBMCs increases the ‘uniqueness’ of the barcode and hence a highly reliable fit can be obtained for a vast majority of the experimental fragments. It should be noted that even when the number of time frames was reduced from ten to a single snapshot, the number of molecules that fit to the human genome with a *P*-value below 10^−6^ was still as high as 79% (30/38), which further demonstrates the potential of competitive binding-based ODM for high-throughput applications. Moreover, the throughput of the ODM assay will also depend on the combination of exposure time and light intensity (Supplementary Figure S5).

The data presented in Figure 4 represents only a small fraction of the human genome. To generalize our results, we performed *in silico* simulations to, *i)* evaluate the impact of fragment size on the *P*-value, and *ii)* assess which parts of the human genome that can be mapped using the competitive binding assay. The results (Supplementary Figure S6) predict that it should be possible to obtain a *P*-value below our threshold of 10^−6^ for all DNA-molecules larger than approximately 275 kb, and in many cases for fragments down to 100 kb.

For the evaluation of mappable regions in the human genome, simulations were made based on the average size (333 kb) and the average *C*_max_ (≈0.81) of the mapped DNA-molecules from Figure 4 (details in Supplementary Methods). In short, the human genome was randomly cut into 333 kb fragments, which were then compared to the complete human genome. All fragments which obtained a *C*_max_ greater than 0.81, excluding the *C*_max_ to the original position of each fragment, are considered to be non-mappable, due to the lack of uniqueness in the barcode compared to the human genome.

**Figure 5.**
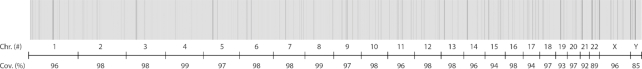
*In silico* evaluation of mappable parts of the human genome. Graphical overview of regions on the human genome possible to explicitly map (gray, 96.8%), as well as regions were DNA-molecules could theoretically match to more than one position with a high match score (*C*_max_) (dark gray, 3.2%).

The results from the simulation (Figure 5) demonstrate that it is possible to map 97% of the human genome with 333 kb DNA molecules, further demonstrating the potential of competitive binding based ODM. The remaining 3%, which are deemed ‘non-mappable’ using a fragment size of 333 kb, represents parts of the human genome that are too similar to be separated via the optical maps. However, even if fragments originating from these regions cannot be exactly placed, the correct position could in many cases be narrowed down to just a few alternatives (see Supplementary Figure S7). Moreover, restricted by the field of view, we are not able to map the largest DNA fragments from the DNA extraction. Using a meandering nanochannel (52) would allow fragments larger than 600 kb in size to be mapped which would mean that the percentage of mappable regions would increase even further. Additionally, with increasing size of the DNA fragments the ability to detect differences over highly variable regions will increase, where one part of the DNA molecule can be used to find a match with a high degree of confidence, while the other part can reveal structural variations.

### Dual labeling for extracting information beyond the DNA sequence

An attractive potential use of competitive binding based ODM is to map other types of information, such as epigenetic marks and DNA damage lesions, to the human genome. As a proof of principle, damage sites in DNA from PBMCs exposed to the chemotherapeutic agent etoposide were labeled *in vitro*, prior to adding YOYO-1 and netropsin to create the sequence specific barcode (see Methods for details). The damage sites were visualized with fluorescently labeled nucleotides and mapped to the human genome via the competitive binding maps.

**Figure 6.**
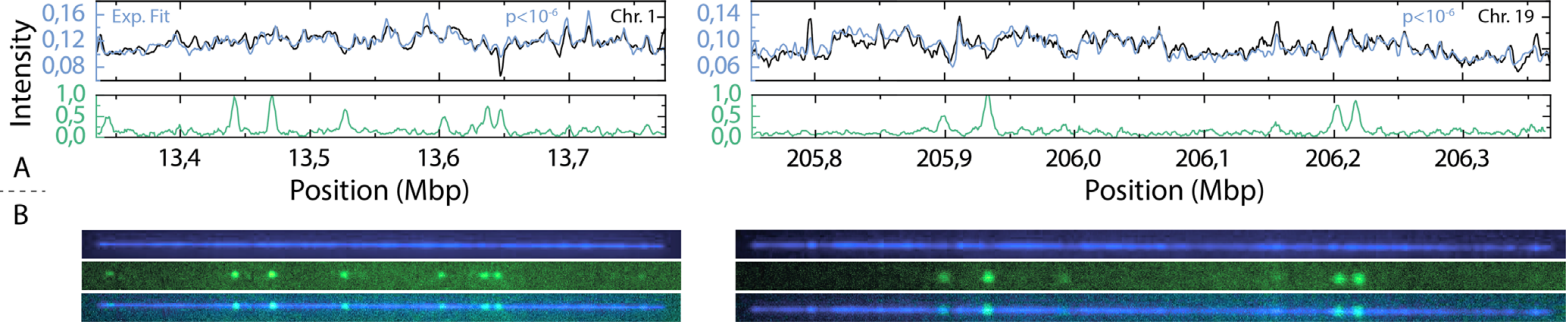
Visualization of DNA damage with corresponding fit to the human genome. Examples of DNA molecules extracted from PBMCs and exposed to the chemotherapeutic agent etoposide. The DNA damage sites are visualized in green (ATTO-647), and the barcode based on the underlying DNA-sequence in blue (YOYO-1/Netropsin). (**A**) Comparison between experimental (blue) and theoretical (black) barcodes, demonstrating excellent overlap and a *P*-value < 10^−6^ (top), as well as the normalized intensity in the 647 channel along the center line of the DNA-molecule displaying the DNA-damage sites (green) (bottom). (**B**) Microscopy images of YOYO-1 channel (blue, top), ATTO-647 (green, center) and overlay of both channels (composite, bottom). The optimal focus will shift slightly for the different emission wavelengths of YOYO-1 and ATTO-647. The left molecule shows an example where the focus was adjusted to the fit the green channel (ATTO-647) and the right molecule an example where the focus was adjusted to fit the blue channel (YOYO-1).

Figure 6 shows that it is possible to simultaneously map DNA molecules to the human genome and visualize damage sites on these molecules. In total 22 out of 25 (88%) mapped molecules (238–608 kb in size) passed the *P*-value threshold of 10^−6^, similar to what was observed when using competitive binding only (95%, Figure 4), which made it possible to combine the competitive-binding assay with enzymatic labels. Besides damage sites on the DNA, other information, such as epigenetic marks, could be visualized in combination with the competitive binding mapping assay. Reduced representation optical methylation mapping (ROM), has been used to simultaneously report on the overall methylation status of a genomic region (31). Moreover, Gabrieli *et al.* showed how the epigenetic mark 5-hydroxymethylcytosine (5-hmC), linked to gene regulation, could be mapped to a specific genomic location using dual labeling, combining the 5-hmC visualization with a nick-labeling enzyme (33). Using the approach presented here, the barcode generated by nick-labeling could be directly replaced with our competitive binding-based barcode, utilizing the already available YOYO-1 channel, previously used for molecule localization only, increasing both throughput and simplicity. More importantly, commercially available mapping platforms such as Irys and Saphyr (Bionano), are equipped with three imaging channels and are thus restricted to the observation of a single mark in addition to YOYO and nick labels. The approach reported here opens up the possibility to study the co-existence of two non-genetic marks. One such application is the simultaneous detection of mC and hmC, two major epigenetic marks that are undistinguishable by traditional bisulfite sequencing.

Regarding optical visualization of DNA damage sites, we foresee that the assay presented here could present diagnostic biomarkers for diseases linked to DNA-repair deficiencies, for example by locating damage sites in transcribed and non-transcribed regions of the genome (53,54). The approach could also allow for more fundamental studies on differences in damage loads across the genome when exposing cells to different damaging agents or hazardous environments. Moreover, as shown here, the damage assay, previously utilizing DNA stretched on glass slides, could also be performed in nanochannels, facilitating homogeneous stretching and simplified quantification.

The use of nick-labeling based ODM has shown great potential for assembly of complex genomic regions or entire genomes, aiding the assembly of contigs obtained from DNA sequencing (12–15). Combining competitive binding based mapping with nick-labeling has the potential to significantly increase the capacity when assembling complex genomes compared to using nick-labeling alone. This is especially important for regions where nick sites are sparse, resulting in genomic ‘blind-spots’. The addition of a sequence specific profile via competitive binding naturally integrates into existing workflows while significantly enhancing the information content of the recorded DNA maps. Dual enzymatic labeling assays have been developed, using three separate channels for aquisition, one for each enzyme as well as one for detecting the contour of the DNA with YOYO-1 (24,55,56). This results in increasing the demand on the imaging setup, and a reduction in throughput due to the additional imaging. Moreover, the preparation protocol complexity is increased, including extra nicking steps, as well as the need to remove all left-over fluorophores between the two different labeling reactions. In contrast, utilizing the YOYO-1 channel not only for detecting the contour of the DNA, but also for acquiring highly specific sequence information, as demonstrated here, the power of ODM could be improved significantly without any additional complexity in either sample preparation or imaging, leaving the throughput unaffected. The experimental integration into existing commercial instrumentation should be straight forward since the YOYO-1 channel is already used for the location of the DNA molecules.

To conclude, we demonstrate the potential of using competitive binding based ODM for high-throughput analysis of the human genome. We show that by optimizing experimental protocols and settings, a vast majority (92%) of the DNA molecules (215–608 kb in size) derived from human blood cells can be located along the human genome. Moreover, *in silico* simulations based on the experimental results illustrate how ∼97% of the human genome can be mapped with high precision at a fragment size of 333 kb. Finally, we demonstrate how competitive binding can be combined with enzymatic labeling, identifying DNA damage sites and pinpointing them to a specific location along the human genome. The principles discussed open up for future multiplexed analysis of genetic and epigenetic marks along the human genome with increased sensitivity and specificity.

## Supplementary Material

gkz489_Supplemental_File
