# Supplementary material for: Enzyme-free optical DNA mapping of the human genome using competitive binding
Source: Nucleic Acids Res. 2019 Jun 5;47(15):e89. doi: 10.1093/nar/gkz489 (PMC6735870; doi:10.1093/nar/gkz489)
Supplement: gkz489_Supplemental_File [file gkz489_supplemental_file.pdf]

Supporting Data

Enzyme-free optical DNA mapping of the human genome using competitive binding

Vilhelm Müller[1], Albertas Dvirnas[2], John Andersson[1], Vandana Singh[1], Sriram KK[1], Pegah Johansson[3], Yuval Ebenstein[4], Tobias Ambjörnsson[2] and Fredrik Westerlund[1]

[1]Department of Biology and Biological Engineering, Chalmers University of Technology, Gothenburg, Sweden
[2]Department of Astronomy and Theoretical Physics, Lund University, Lund, Sweden
[3]Clinical Chemistry, Sahlgrenska University Hospital, Gothenburg, Sweden
[4]School of Chemistry, Center for Nanoscience and Nanotechnology, Center for Light-Matter Interaction, Raymond and Beverly Sackler Faculty of Exact Sciences, Tel Aviv University, Tel Aviv, Israel

Supporting Tables

Table S1: Overview of bacterial artificial chromosomes (BACs) used in the study.

BAC (ID)	Chromosome	Position (Mb)	Estimated Size* (kb)	# Molecules Analyzed
**2330P18 (P18)**	22	45.71**	123	44
**3149J19 (J19)**	22	45.74**	130	34
**936J21 (J21)**	X	147.87	203	47
**322C17 (C17)**	6	28.84	159	32
**495H8 (H8)**	6	32.44	138	30

** After restriction with NotI ** Partially overlapping sequence, J19 follows P18 on chromosome 22*

Supporting Figures

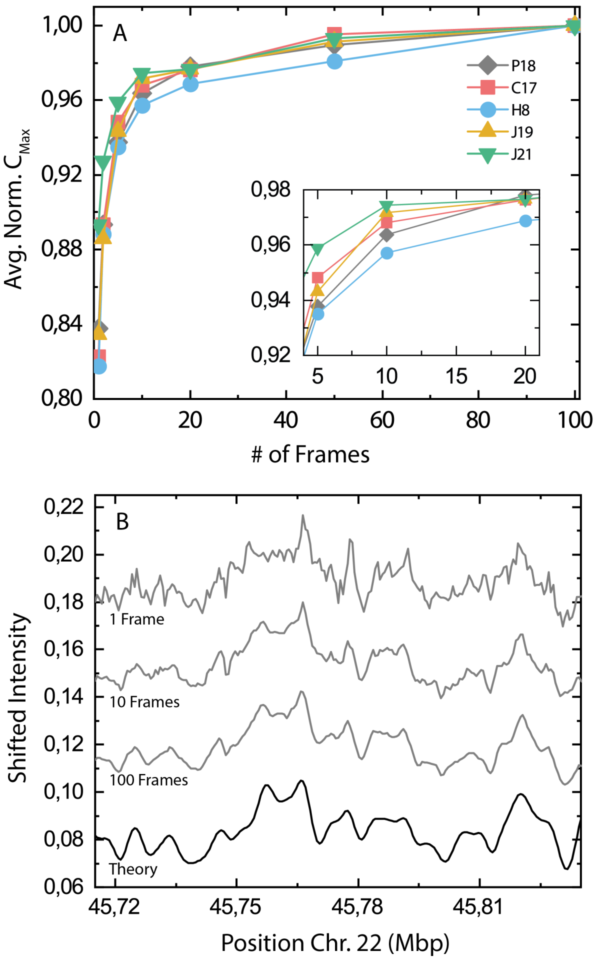

Figure S1: **The effect on match scores (C_{max}) with varying number of acquired frames.** *A) Average normalized C_{max} values for different number of frames for BACs P18, C17, H8, J19 and J21 when fitted to their corresponding theoretical barcode B) Comparison of theoretical P18 barcode (black) with experimental P18 barcodes consisting of different number of frames as indicated (gray).*

With increasing number of time frames the signal to noise ratio decreases, and hence the precision of the obtained fit to the theoretical reference increases. However, even when using a single time frame 83% of the maximal score is obtained, and for 10 frames the number is as high as 96%.

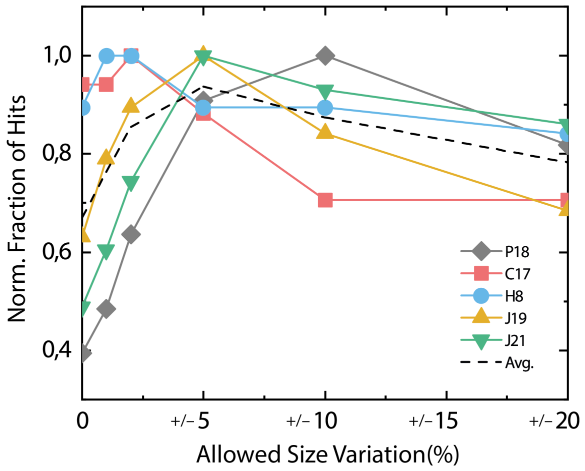

Figure S2: **Fraction of correct hits as a function of allowed size variation.** *Experimental barcodes from BACs P18, C17, H8, J19 and J21 mapped to the human genome with different amount of size variation allowed (1% steps).*

The optimal amount of allowed size variation for the five examined BACs was always between 2-10%, with an overall optimal value of 5%. As expected, the fraction of correctly placed fragments initially increased as more stretching was applied, and later decreased as more attempts of stretching increases the probability that the experimental barcodes map well also to incorrect positions along the human genome.

The sensitivity to amount of size variation allowed for an experimental barcode will vary depending on the degree of AT/GC variations in the underlying DNA sequence, which could explain the differences for the different BACs. Moreover, minor experimental errors in pinpointing the correct extension of the DNA based on the reference lambda-DNA molecule could also affect the results. The precision increases with increasing number of time frames, since an average extension can be better estimated.

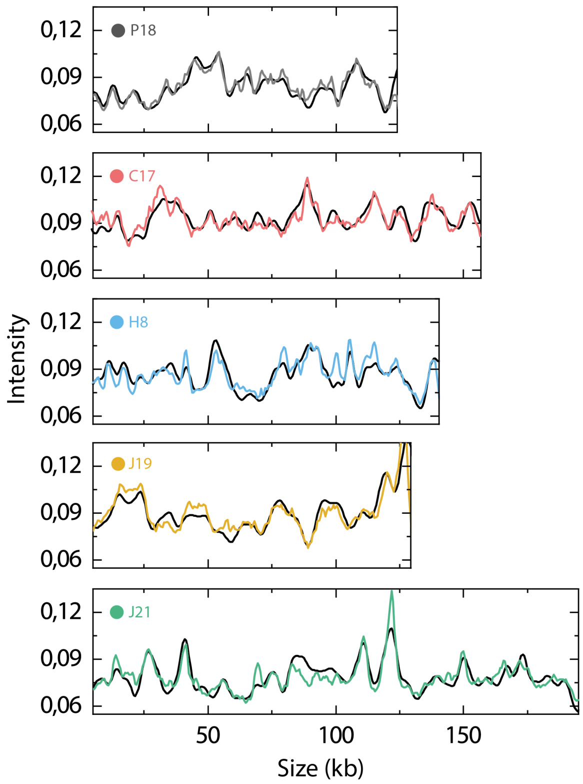

Figure S3: **Examples of fits for individual experimental BAC barcodes to the corresponding correct position along the human genome.** *All barcodes were mapped using 10 frames and up to 5% stretch (1% steps).*

For all 5 BACs individual fragments could be mapped to the genome with high precision. As expected, the signal to noise ratio is larger than for the consensus barcodes of the BACs (Figure 2, main text). However, this does not seem to affect the possibility to map the fragments to the correct position in the human genome to any large extent.

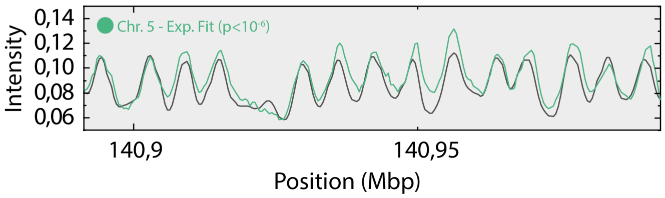

Figure S4: **Repetitive region found in experimental fragment when matched to human genome.** *Zoomed in version of repetitive region in Figure 4C (main text). The theoretical barcode is shown in black and the experimental barcode in green.*

The possibility of ODM to acquire long range sequence information is a great advantage compared to traditional sequencing techniques. Using ODM, repeats can be quantified directly from the barcode, exemplified by the repetitive region of the GC rich exons of the Protocadherin Alpha gene cluster, mapped in Figure S4.

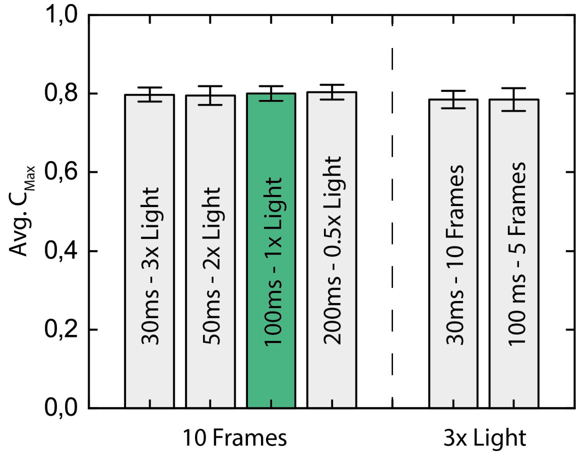

Figure S5: **Impact of exposure time, light power and number of frames on data quality.** *Average C_{max}-values with corresponding standard deviation (N=20) for experimental J21 BAC barcodes when compared to its corresponding theoretical barcode with different amounts of light, exposure time and time frames. The green bar represents the general settings used for acquiring the data in study. The acquisition time for the camera to acquire ten images with 30 ms exposure is approximately the same as the time to obtain five images with 100 ms exposure.*

In order to evaluate the possibility of increasing the throughput in future applications, experiments were made with lower exposure time per frame, compensated by higher intensity of the excitation light. The results show that exposure times down to 30 ms can be used when compensated by increased light exposure, reducing the acquisition time by a factor 2 compared to when using 100 ms exposure. The results also show that the number of time frames can be reduced when using a higher light intensity, without any large effect on the data quality.

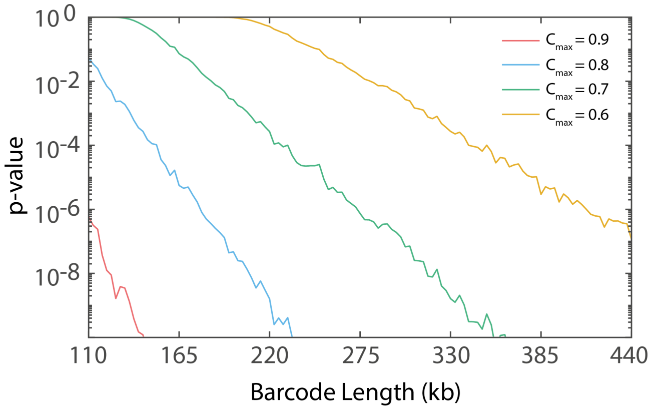

Figure S6: **Simulated p-values expected for different lengths and match scores.** *The expected p-value for match scores* C_{max} *of 0.9, 0.8, 0.7 and 0.6 for DNA molecules of varying length (kb).*

The data presented in Figure 4 (main text) represents only a small fraction of the human genome. To generalize our results and study the effect of fragment size on the p-value of a fit between an experimental barcode and the human genome, *in silico* simulations were performed using randomized theoretical fragments of varying sizes (Figure S6, details in Supporting Methods). For each size (steps of approximately 3 kb), one thousand randomized theoretical barcodes, preserving the statistical properties of the human DNA, were compared to the randomized theoretical version of the human genome. The distribution of C_{max} scores for each size was then used in order to predict the level of significance (p-value) that would be obtained for an experimental barcode with different C_{max} scores (0.6-0.9).

The C_{max} obtained when comparing an experimental barcode to the human genome is typically in the range of 0.7-0.9. The results shown in Figure S6 predict that it should be possible to obtain a p-value below our threshold of 10^{-6} for all DNA-molecules larger than approximately 275 kb, using a C_{max} of > 0.7. Moreover, with a C_{max} of 0.9 we can successfully map fragments down to 110 kb or even smaller, demonstrating the high discriminatory power of the obtained barcodes.

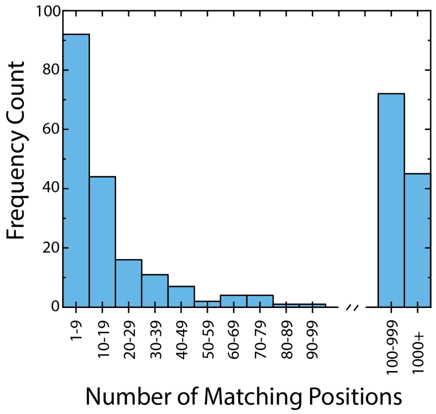

Figure S7: **Number of matching positions for none-mappable regions within the human genome.** *The histogram depicts the number of additional positions to which each DNA fragment, deemed non-mappable in Figure 5 (main text), matches with a greater* C_{max} *than 0.81.*

Many of the none uniquely mappable regions only show a high degree of similarity to a few additional positions of the human genome (Figure S7). Hence, the correct match position for an experimental barcode from a none-mappable region can be narrowed down to only a few positions within the entire human genome.

Supporting Methods

Process experimental data

In this section we describe how the output of the nanofluidics-based fluorescence imaging experiments was processed using custom written MATLAB software. The workflow can be summarized: Imaging output (Movie) $\rightarrow$ kymograph $\rightarrow$ barcode.

Extract kymographs

As output from the nanofluidics experiments, we obtain three dimensional arrays (movies) of fluorescently stained DNA molecules. These movies are then processed following the outline in [1] in order to generate kymographs. The method is summarized in Algorithm 1, and the steps are explained in more detail below.

```
input  : movie
output: kymograph
Rescale movie;
Amplify movie;
Rotate movie;
Find the angle of molecules in the movie;
Find foreground;
Locate molecule along the nanochannel;
Average over 3 pixel width to get a kymograph;
```

Algorithm 1: Generate kymographs from movies

1. Rescale movie values (which are usually integers `int16`) to values in the interval $[0, 1]$,

$$A(i,j,k) := \frac{A(i,j,k) - \min(A)}{\max(A) - \min(A)} \tag{1}$$

2. Amplify the movie by convolution with an amplification kernel (3 by 3 square with zero at the center), i.e. with the matrix K

$$K = \begin{pmatrix} 0.125 & 0.125 & 0.125 \\ 0.125 & 0 & 0.125 \\ 0.125 & 0.125 & 0.125 \end{pmatrix} \tag{2}$$

   Then the convolution is defined as

$$A_{amplified}(i,j,k) = (K * A)(i,j,k) = \sum_{s=-1}^{1} \sum_{t=-1}^{1} \sum_{l=-1}^{1} K(s,t) A(i-s, j-t, k-l) \tag{3}$$

3. Find the angle of the movie:

(a) Average the movie over time frames $k = 1 \ldots N$.

$$A_{averaged}(i,j) = \frac{1}{N} \sum_{k=1}^{N} A_{amplified}(i,j,k) \tag{4}$$

(b) Use morphological top hat filtering with a disk D of radius 12 pixels to correct uneven illumination for the dark background, i.e.

$$A_{corrected}(i,j) = A_{amplified}(i,j) - imopen(A_{amplified}, D)(i,j) \tag{5}$$

where *imopen* is a morphological image opening operation first applying erosion and then dilation on the image.

(c) Find edges in $A_{corrected}$ using the Sobel method, which uses Sobel approximation to the derivative, and returns edges at those points where the gradient of $A_{corrected}$ is maximum.

(d) Apply a standard Hough transform. It uses parametric representation for the line

$$\rho = i \cdot \cos(\theta) + j \cdot \sin(\theta), \tag{6}$$

i.e. each matrix element (i,j) is converted to a ρ and θ matrix. The angles are fixed to be in the range $[-90 : .01 : 89.99]$. The optimal angle is then the highest peak in this matrix.

(e) Use the detected angle to rotate the movie. Consequently, the nanochannel with the molecule will be oriented vertically. After the rotation, there might be some pixels at the edges of the rotated movie that do not correspond to pixels in the original movie. We refer to these as undefined pixels.

4. Find foreground of the movie (simple amplification and thresholding);

(a) Compute the mean and standard deviation of the intensity of the pixels of the movie. Use these to substitute the undefined pixels with pseudo-random (`rng(rng(0, 'twister'))` in Matlab) values.

(b) Amplify the movie by using convolution with a kernel whose matrix has elements $((x^2 + y^2)^{-0.25})$ for $x = -2, \ldots, 2$, $y = -2, \ldots, 2$. Normalize and square the output movie;

(c) Further amplify the movie by using convolution with the vector $\begin{bmatrix}-1.5 & -0.25 & 1.25 & 2 & 1.25 & -0.25 & -1.5\end{bmatrix}$. Normalize the output movie;

(d) Average the movie along the time-frames dimension and along the rows dimension, leaving the averaged column fluorescence profile. Find local extrema in this profile. From these, choose the extrema with intensities higher than mean+3 standard deviations of the intensities. The columns between minimas on the left and right of this maxima are labeled as signal columns;

(e) To find foreground values in these columns, we define a threshold for intensity which is the minimum of Matlab's `graythresh()` (which is computed for all non-signal column pixels) and `mean() + 3std()` (which is computed only for those values that are below the `graythresh()` value);

(f) Find pixels that are identified as signal in all time-frames (or in at least 20 time-frames, if there are more than 20 time-frames). Label the columns that have less than 20 of such pixels as non-signal columns;

5. Locate the molecule along the channel;

(a) Extract intensity values for the signal columns of the time-averaged rotated movie;

(b) Apply the Gaussian filter with $\sigma = 10$ pixels, and kernel of size $\begin{bmatrix} 50 & 3 \end{bmatrix}$ pixels;

(c) Run Matlab's `findpeaks()` to detect molecule center index;

(d) Find the molecule row and column coordinates in the channel (to that end we fit a function $a + f \cdot (\tanh((x-b)) - \tanh((x-c)))$);

6. Average the molecule over 3 columns and with 100 row padding on the edges of the molecule to generate a kymograph.

Align kymographs

To compensate for small thermal fluctuations of the DNA inside the nanochannels, features in the kymograph need to be aligned. This is done using the alignment method NRAlign, introduced in [2], which in turn is an improvement of the WPAlign method, introduced in [1]. The steps of the algorithm are described in Algorithm 2.

input : unalignedKymo
output: alignedKymo

Pre-process by performing shift based alignment;
Smooth the shift-aligned kymograph;
Find k features;
Find stretch factors;
Apply horizontal stretching;

Algorithm 2: Align kymographs

1. Shift align kymograph;

   (a) Apply a Gaussian filter of $\sigma = 2$ pixels, and kernel of size $[1, 10]$ pixels;

   (b) Use cross correlation (allowing 3 pixel shift per row) as a measure to estimate the shift of kymograph rows with respect to the first row;

   (c) Shift rows of the `unalignedKymo` using the estimated shifting values;

2. Smooth the shift-aligned kymograph;

   (a) Apply a Gaussian filter of $\sigma = 2$ pixels, and kernel of size $[10, 10]$ pixels to the shift aligned kymograph;

   (b) Apply a Laplacian of Gaussian filter of $\sigma = 2$ pixels and size $[2, 6]$ pixels;

3. Find k features. The maximum number of features saught is $\lceil \frac{n_c}{11} \rceil$ where n_c is the number of columns in the kymograph. See [2] for details how these features are computed;

4. Find stretch factors. We compute a matrix describing how much each pixel should be stretched to align all k features;

5. Iterate through the rows of the kymograph. Aligned rows are created by linearly stretching using the cumulative sum of the stretch factors of each pixel;

Limit number of time-frames

Kymographs have varying number of time-frames (rows), depending on how many snapshots of the molecule are used. We study the efficiency of the method based on always selecting a fixed number of timeframes, n_k. This reduces the set of kymographs to only those kymographs that have at least n_k rows, and in particular, we have a set of kymographs $\hat{K}$, with

$$\hat{K}_i = \{K_i(x, y) \mid x \in \overline{1, n_k}, y \in \overline{1, m}\}. \tag{7}$$

From kymographs to time-averaged barcodes

To compute time-averaged kymographs, we need to first detect the edges in the kymograph. Once the barcodes and their bitmasks are computed, we can generate consensus barcodes or compare to theory, and here we use the same methods for that as in [3].

Edge detection in kymographs We use Otsu's method (`multithresh` in Matlab), morphological operations (`imclose`), and component analysis (`bwconncomp`) to separate the foreground from the background. We then find the longest contiguous foreground component in each time frame (row) represented in the kymograph. The output is a two dimensional vector, which for each time-frame (row) stores two indices: one column where the molecule starts and one where it ends.

Barcodes Molecule start indices are averaged to estimate the first pixel position of the molecule, and molecule end indices are averaged to estimate the last pixel position of the molecule. The kymograph is then time averaged, and the pixels from the estimated first pixel position to the estimated last pixel position represent a barcode. The kymograph values outside the molecule pixels (signal region) are used to estimate the mean and standard deviation of the background by fitting a Gaussian using `fitdist()` function in Matlab.

Bitmasks A bitmask is a binary vector associated to the barcode, and its values are ones except at the edges of the barcode. The number of pixels at the edges is chosen based on point spread function, which is $300nm$, and pixel to nano-meter convertion ratio of the experiment, which is $130nm/px$, and a $\Delta = 3$. The number of pixels which are zeros at the edges of the bitmask is then $3 \cdot 300/130 \approx 7$ pixels.

In-silico simulations

We here use in-silico simulations in order to i) compute a DNA barcode for a known DNA sequence, ii) simulate DNA barcodes by using randomization iii) compute p-values for matching experimental barcodes to theory, and iv) estimate how much of the human genome we can cover using this method.

Theory generation

Generating theory barcodes DNA sequences of the 24 human chromosomes (GRCh38.p7 dataset) in .fa.gz format were downloaded from RefSeq, NCBI Reference Sequence Database (`ftp://ftp.ncbi.nlm.nih.gov/genomes/Homo_sapiens/`, and are summarized in table SM1

Table SM1: Theory files

**url**	**version**	**files**
`.../ARCHIVE/ANNOTATION_RELEASE.108/Assembled_chromosomes/seq/`	`ANNOTATION_RELEASE.108`	24

DNA sequences were converted into theoretical barcodes as described in [3]. For completeness, the method's steps are summarized here:

1. A set of input parameters are provided, described in Table SM2. The competitive binding model used here, which we call 'literature', uses parameter values as described in [3]. `concDNA, concY, concN` are the concentrations of DNA, YOYO-1 and netropsin. `psfSigmaWidth` is the width of point spread function in nanometers. `meanBpExt` is the scaling factor (nm/bp) of the nanometer to base-pair extension. `pixelWidth_nm` is the nm to pixel conversion ratio.

Table SM2: Parameters for theory generation

**Parameter**	**Value**
model	'literature'
concDNA	0.2 μM
concY	0.02 μM
concN	6 μM
psfSigmaWidth	300 nm
pixelWidth_nm	130 or 208 nm/px
meanBpExt_nm	0.3 nm/bp
isLinearTF	1

2. We use λ-page DNA (48502 bp), and the total concentrations of YOYO-1, *concY* (C_Y) and of netropsin *concN* (C_N) to compute their free concentrations. This is done by first computing what fraction of the DNA (base pairs) on average that would be occupied by netropsin (alternatively YOYO-1) by computing $f_N(C_N, C_Y)$

$f_Y(C_N, C_Y)$). These are just the averages of netropsin (YOYO-1) binding probabilities along the DNA. We then define a two variable function

$$\begin{pmatrix} g_1(C_1,C_2) \\ g_2(C_1,C_2) \end{pmatrix} = \begin{pmatrix} C_N \\ C_Y \end{pmatrix} - \begin{pmatrix} C_1 f_N(C_1,C_2) \\ C_2 f_Y(C_1,C_2) \end{pmatrix} \cdot C_{DNA} \cdot 0.25 \tag{8}$$

which we minimize to find the free concentrations of Netropsin and YOYO-1, C_1 and C_2,

$$\min_{C_1,C_2} \left((g_1(C_1,C_2))^2 + (g_2(C_1,C_2))^2 \right) \tag{9}$$

For this we use multidimensional unconstrained nonlinear minimization (Nelder-Mead), using MATLAB's in-built function `fminsearch` with initial parameters $concN$ and $concY$.

3. Any undefined nucleotides are changed into random letters using Matlab's command `randseq`;
4. If the sequence is assumed to be linear, add $N = 10000$ base-pairs to the ends of the sequence;
5. If the sequence is longer than 500000 base-pairs, cut it into smaller parts of length 200000 to save memory with added 3000 base-pairs at the ends;
6. For each of these fragments, compute the probability that YOYO-1 is bound to a base-pair i, $p(i)$;
7. Convolve these fragments with a Gaussian and (if the sequence was cut into smaller fragments) stack these back together to one long sequence, to get a barcode of the form

$$\tilde{I}(k) = (\mathbf{p} * \phi)(k) = \sum_{i=0}^{k_{\max}-1} p(i) \cdot \phi(i-k), \; 0 \le k \le k_{\max} - 1 \tag{10}$$

8. Remove the extra base-pairs at the ends if the sequence was linear ($isLinearTF = 1$);
9. Convert from base-pair resolution to pixel resolution using a moving average window;

Stretching theory The theoretical barcode of the whole human genome in base-pair resolution takes a large amount of space and contains redundant information, since when doing the comparisons, we are interested in pixelated theory. Therefore instead of saving the barcode with base-pair resolution, we save it with pixel resolution.

This provides us with a challenge, because theory with base-pair resolution depends on nm to bp extension of the experiments. Since this can vary from experiment to experiment, to cover all possibilities, we would need to compute the theory for a very large number of possibilities.

Instead, we make an observation that a convolution of two Gaussians of width's σ_1 and σ_2 is still a Gaussian, with width

$$\sigma = \sqrt{\sigma_1^2 + \sigma_2^2} \tag{11}$$

Using this observation, we first compute the pixelated theory for one pre-chosen nm/bp ratio (this should not be smaller then the largest nm/bp expected in the experiments). In this case we chose it to be 0.3 nm/bp. This will give us a theory barcode with a convolution with a Gaussian of σ pixels width. However, if the actual nm/bp is different, then we need to compress the theory barcode by s, i.e. all the Gaussians in the convolution should have width $\sigma_1 = \sigma \cdot s$, $s < 1$. This is not equal to σ unless $s = 1$.

However, we can use our observation about convolution of two Gaussians to correct σ. Since $\sigma_1 = \sigma \cdot s$, we just need to convolve with a Gaussian of unknown width σ_2, which we find from equation 11 to be

$$\sigma_2 = \sqrt{\sigma^2 - (\sigma \cdot s)^2} \tag{12}$$

Stretching when comparing experiments to theory A stretching factor of 5% at increments of 1% was used when comparing experiments to theory. We stretched the experiments to all the possible versions using these stretching factors. For stretching, we used Matlab's `interp1` function, which returns interpolated values at specific query points using linear interpolation.

Randomized barcode

In here, we use a simpler approach for generating randomized barcodes than in [3]. Our present approach is described by Algorithm 3. Briefly, to simulate a barcode of length *lenBar* we first compute *lenBar* normally distributed (mean = 0, standard deviation = 1) random numbers. We then convolve this vector with a Gaussian kernel of a given point spread function with width psf (see Algorithm 4).

input : lenBar, pixelWidth_nm, psfSigmaWidth_nm
output: simBar

psf ← psfSigmaWidth_nm/pixelWidth_nm;
rand ← $(X_1, \ldots, X_{\text{lenBar}})$, where $X_t \sim \mathcal{N}(0, 1)$;
simBar ← convolve_bar(rand, psf) ;

Algorithm 3: Function sim_bar for computing a randomized barcode `simBar`

input : bar, sigma, hsize
output: barOut

ker ← circshift(images.internal.createGaussianKernel(sigma, hsize), round(hsize/2));
multF ← conj(fft(ker'));
barOut ← ifft(fft(bar).*multF);

Algorithm 4: Function `convolve_bar` for computing a convolution of random vector `bar` with a Gaussian kernel.

We expect that this method provides a realistic representation of a barcode at the pixel level, since at the base-pair level we do not expect to have long range correlations unless there are repetitive/homogenous regions. See figure SM1 for example of a randomized barcode using our new approach.

Slow computation of p-values

In this section we explain how the p-values can be computed. We do not use this method, but rather present it here for reference (in the next subsection we explain how we speed up the method). Parameters for computing p-values are given in Table SM3. For example, `strFac` describes the stretching allowed when comparing experiments to theory, which here is 5% with 1% increments.

For a single experimental barcode, the scheme of how to generate the p-value follows [3], and is here presented in Algorithm 5.

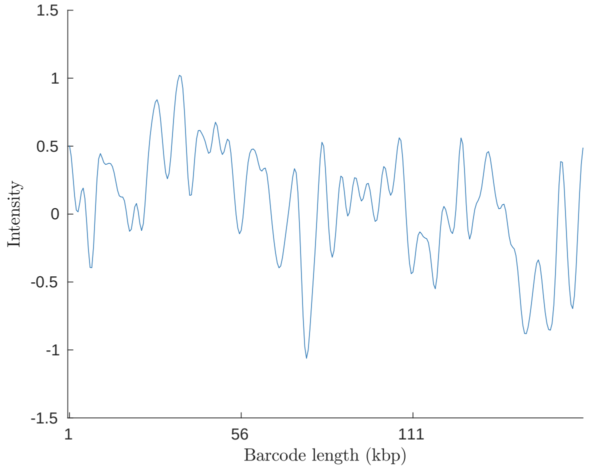

Figure SM1: Randomized barcode

Table SM3: Table with p-value generation parameters

**Parameter**	**Value**	**Description**
strFac	0.95:0.01:1.05	Stretching factors
cMaxVal	[0,1]	Maximum match score
barLen	[200:1100]	Length of the experimental barcode in px
numRnd	1000	Number of random barcodes
pixelWidth_nm	130/208	Pixel width in nm/px
psfSigmaWidth_nm	300	Point spread function in nm
lenLong	5610000/3981312	Theory length in px

input : cMaxVal,strFac, lenBar, lenLong, pixelWidth_nm, psfSigmaWidth_nm, numRnd
output: pVal

randLong← $sim\_bar(lenLong, pixelWidth\_nm, psfSigmaWidth\_nm)$;
for $i \leftarrow 1$ **to** $length(strFac)$ **do**
  $data[i,:] \leftarrow zeros(length(strFac), numRnd)$;
  for $j \leftarrow 1$ **to** $numRnd$ **do**
    rand← $sim\_bar(round(lenBar \cdot strFac[i])), pixelWidth\_nm, psfSigmaWidth\_nm)$;
    data[i,j] ← max compute_pcc(rand,randLong) ;
  end
end
maxCCVals ← $max(data[i,j], 1)$;
evdPar ← compute_distribution_parameters(maxCCVals,'functional',barLen/5);
pVal ← compute_p_value(cMaxVal, evdPar,'functional');

Algorithm 5: Function `compute_p_value` to compute p-value for a barcode that has `cMaxVal` maximum match score against the theory.

Briefly, we first simulate a long barcode of length equal to the length of the theory, which is `lenLong` pixels. Then for each stretching factor, we compute `numRnd` randomized barcodes. The length of these barcodes depends on the stretching factor. Then we compute the maximum score for each of these randomized barcodes against the theory. The scores for all positions when comparing a short barcode against a long barcode is computed by function `compute_pcc()`, which computes the match scores by sliding the shorter barcode along the longer barcode, and in both directions (see [3]). The resulting matrix of match scores is averaged over the stretch factors, and distribution parameters of a functional form is computed using a function `compute_distribution_parameters()`. Finally `compute_p_value` is used to compute the p-value given a match score `cMaxVal`.

The main differences between this method and that in [3] is that we change the way we generate randomized barcodes, we simulate the longer barcode (this is needed for the section to follow), and we add stretching.

Fast computation of p-values

Here we describe our new method for computing p-values as used in the main text. Our new method is considerably faster than the method presented in the previous subsection. The speed-up comes from fact that we pre-generate a database for a range of different barcode lengths, and an average bp/nm extention ratio. This database is then used to skip steps 2-8 in Algorithm 5.

To generate the p-value database, we use the parameters of Table SM3, as well as two additional parameters listed in Table SM4. The algorithm we use to compute a database is given in Algorithm 6. In this method we perform similar calculations to those in steps 2-8 of Algorithm 5. We compute a randomized long barcode. Then instead of using different stretching factors in the first loop, we use lengths of barcodes directly in the first loop. The inside loop differs from Algorithm 5 in that the first parameter to `sim_bar` is the length of barcode i. The output of this calculation is a database file data, which can be saved as `data.dat`, or in a simple text format, as we save it to `PVALTOT.txt`.

Table SM4: Table with additional database generation parameters

Parameter	Value	Description
lenMin	150 px	Minimum length for which to generate match scores
lenMax	1100 px	Maximum length for which to generate match scores

```
input  : lenLong, pixelWidthnm, lenMin, lenMax, psfSigmaWidh
output: data (also PVALTOT.txt file)
randLong← sim_bar(lenLong, pixelWidth_nm, psfSigmaWidth_nm);
for i ← lenMin to lenMax do
    data[i,:] ← zeros(1,numRnd) ;
    for j ← 1 to numRnd do
        rand← sim_bar(i, pixelWidth_nm, psfSigmaWidth_nm);
        data[i,j] ← max compute_pcc(rand1,rand2) ;
    end
end
```

Algorithm 6: Function `pregenerate_pvalue_db`, used to compute a database for fast p-value calculation.

Once the database is computed, we can use it to compute a p-value for barcode of length l. Given this length, we compute the different lengths of the barcodes we can have when including stretching factors `strFac`. This gives

us l_{vec} different lengths, i.e.

$$l_{vec} = \left\{ \lfloor l \cdot strFac_i \rfloor \mid i = \overline{1, length(strFac)} \right\} \tag{13}$$

We use these to get a sub-matrix of the pre-generated database, defined as

$$d = data[l_{vec}, :] \tag{14}$$

Finally, we average the rows to get numRnd maximal correlation coefficients.

$$\text{maxCCVals} = \frac{1}{|l_{vec}|} \sum_{i=1}^{|l_{vec}|} data[l_{vec}(i), :] \tag{15}$$

We then proceed with step 10 of Algorithm 5.

Simulation of p-values for different lengths (Supporting Figure S6)

As before, we have fixed nm/bp ratio 0.2363, and stretching of 5% at 1% increments. We then use lengths from 200 to 800 pixels (taken every 5 pixels), i.e. 110kb to 440kb. Then, for a given length, we take all the match scores from the database for the length +-5%. We take the maximum over the dimension of different lengths. This gives us 1000 match scores.

For these scores, we compute the parameters of the extreme value distribution, and then find a threshold of the p-value for which the match score falls bellow one of 4 cases: $0.9, 0.8, 0.7, 0.6$. We finally plot the barcode lengths versus the p-value threshold for each of these 4 cases. The results can be seen in Supporting Figure S6.

Evaluation of mappable parts of the human genome (Figure 5)

We ran evaluation using nm to bp ratio of 0.269 with the average size 689 pixels, which gives approximately 333 kilo base-pairs (more precisely $689 \cdot \frac{130}{0.269}$).

Table SM5: Parameters for estimating the coverage of human DNA by optical maps

Parameter	Value	Description
lenBarcodes	689	Length of barcode fragments
newNmBp	0.269	nm to bp ratio
averageCC	0.81	Match score value used as a threshold for what is mappable.

The genome is cut into fragments. The full comparison would require us to cut the genome into all possible L-mers. However, mainly to reduce the computational time, and because we do not expect this to have significant effect on the overall coverage percentage, we cut the genome into non-overlapping fragments. Each of the 24 chromosomes is cut into N_i fragments of equal lengths $lenBarcodes = 689$ (where i is the number of the chromosome). There are thus some pixels at the right ends of chromosomes that are not included in the calculations, since we only consider non-overlapping fragments.

These fragments are then mapped against the non-overlapping part of the human genome, i.e. the whole human genome with a deletion. To compare the fragment against the theory, we use Pearson correlation coefficient C as a match score. The maximum correlation C_{max} is saved for each fragment. All fragments which obtained a maximum correlation coefficient greater than 0.81 to any part of the human genome are considered to be non-mappable, due to the lack of uniqueness in the barcode. The results can be seen in Figure 5 (main text).

References

[1] Noble, Charleston, et al. A fast and scalable kymograph alignment algorithm for nanochannel-based optical DNA mappings.c PloS ONE, 2015, 10.4: e0121905.

[2] Nordanger, Henrik. Gene-ID Using Simultaneous DNA Barcoding and Enzymatic Labeling. 2017.

[3] Dvirnas, Albertas, et al. Facilitated sequence assembly using densely labeled optical DNA barcodes: A combinatorial auction approach. PloS ONE, 2018, 13.3: e0193900.
